# Higher-order risk preferences in social settings

**DOI:** 10.1007/s10683-017-9541-4

**Published:** 2017-09-08

**Authors:** Timo Heinrich, Thomas Mayrhofer

**Affiliations:** 1 Durham University Business School, Mill Hill Lane, Durham, DH1 3LB UK; 2 School of Business Studies, Stralsund University of Applied Sciences, Stralsund, Germany; 3 Massachusetts General Hospital and Harvard Medical School, Harvard University, Boston, MA USA

**Keywords:** Experiment, Risk aversion, Prudence, Temperance, Communication, Responsibility, C91, C92, D70, D81

## Abstract

We study prudence and temperance (next to risk aversion) in social settings. Previous experimental studies have shown that these higher-order risk preferences affect the choices of individuals deciding privately on lotteries that only affect their own payoff. Yet, many risky and financially relevant decisions are made in the social settings of households or organizations. We elicit higher-order risk preferences of individuals and systematically vary how an individual’s decision is made (alone or while communicating with a partner) and who is affected by the decision (only the individual or the partner as well). In doing so, we can isolate the effects of other-regarding concerns and communication on choices. Our results reveal that the majority of choices are risk averse, prudent, and temperate across social settings. We also observe that individuals are influenced significantly by the preferences of a partner when they are able to communicate and choices are payoff-relevant for both of them.

**Electronic supplementary material:**

The online version of this article (doi:10.1007/s10683-017-9541-4) contains supplementary material, which is available to authorized users.

## Introduction

Much of the economic research on behavior under uncertainty has focused on risk aversion. However, over the course of the last decades it turned out that higher-order risk preferences like prudence and temperance also impact many decisions. Their importance has first been pointed out with respect to saving decisions (e.g. Leland 1968; Sandmo 1970). Subsequently it has been shown that higher-order risk preferences also affect behavior in various fields, such as auctions (e.g. Esö and White 2004; Kocher et al. 2015), bargaining (e.g. White 2008; Embrey et al. 2016), public good provision (e.g. Bramoullé and Treich 2009), or medical decision making (e.g. Eeckhoudt 2002; Felder and Mayrhofer 2014, 2017). This study makes the first attempt to assess higher-order risk preferences in social settings under controlled conditions. In particular, we focus on two aspects that have been found to influence behavior in various experiments (often contrary to standard economic theory): communication and concerns about the payoff of others. In a laboratory experiment we elicit higher-order risk preferences of individuals and systematically vary how an individual’s decision is made (alone or while communicating anonymously with a partner) and who is affected by the decision (only the individual or the partner as well).

In the framework of expected utility theory risk aversion is captured by a negative second derivative of the utility function. Prudence refers to a positive third derivative (Kimball 1990) and temperance to a negative fourth derivative of the utility function (Kimball 1992). Most of the commonly used utility functions imply mixed risk aversion, which means that the derivatives of the utility functions exhibit alternating signs (see Brocket and Golden 1987; as well as Caballé and Pomansky 1996). These utility functions imply risk averse as well as prudent and temperate behavior. In typical life-cycle models of consumption, prudence implies that an individual saves more if the risk of future income increases (also called “precautionary saving” which leads to more precautionary wealth) while temperance is a necessary condition that introducing an independent unfair background risk reduces the investment in risky assets (a necessary and sufficient condition is a combination of higher-order risk preferences called risk vulnerability, see Gollier and Pratt 1996).^1^ However, empirical estimates for the fraction of saving that is precautionary are extremely diverse, ranging from close to zero to greater than 50% (see Geyer 2011, for an overview). In their critical review of the topic, Carroll and Kimball (2008) conclude that estimates of precautionary wealth are sensitive to elicitation procedures and subject to problems from unobserved heterogeneity.

Therefore, recent studies have analyzed the prevalence of higher-order risk preferences via laboratory experiments in which individuals decide privately over lotteries that only affect their own payoffs (see Tarazona-Gómez 2004; Deck and Schlesinger 2010, 2014, 2016; Ebert and Wiesen 2011, 2014; Maier and Rüger 2012; Krieger and Mayrhofer 2012, 2017; Noussair et al. 2014; Haering et al. 2017). Most of these experiments are based on the model-independent and non-parametric definition of higher-order risk preferences introduced by Eeckhoudt and Schlesinger (2006). They find that a majority of choices are risk averse, prudent, and temperate—the only exception is the study by Deck and Schlesinger (2010) which finds more intemperate than temperate behavior.

In a recent study, Noussair et al. (2014) experimentally elicit higher-order risk preferences from a representative sample of the Dutch population and observe most choices to be risk averse, prudent, and temperate. They also correlate individual lottery choices with individual field behavior. Interestingly, Noussair et al. (2014) do not observe any correlation of risk aversion with financial decisions in the field. However, in line with theoretical results, they observe more prudent individuals to have less credit card debt and more temperate individuals to have less risky investment portfolios. These real world decisions, however, are most often not made individually but in the social settings of households or organizations. That means, they affect more than one person or are made after consulting with others. In this respect, the settings in which they are made differ from the settings that have been studied experimentally thus far.^2^

In this paper, we study the social dimension of higher-order risk preferences. To limit confounding gender effects we focus on the behavior of male subjects only. We find that the majority of subjects are risk averse, prudent, and temperate individually and across social settings. Moreover, other-regarding concerns and the ability to communicate with a partner do not impact (higher-order) risk preferences per se. However, we observe a significant influence of the partner on the individual’s decisions with respect to risk aversion and prudence when subjects are able to communicate and the decision affects both.

The rest of the paper is organized as follows. Section 2 discusses related papers on second-order risk aversion in social settings and presents our hypotheses. Section 3 describes the experimental design and procedure. Section 4 presents the results and Sect. 5 concludes the paper.

## Related literature and hypotheses

Several empirical studies have analyzed decision making under uncertainty in social settings without considering higher-order risk preferences. One stream of the literature starting with Stoner (1961) compares decisions made by groups to decisions made by individuals. Since then, hundreds of studies have examined group decisions and found that group members move to the more extreme points when making decisions; that is, they exhibit risky shifts or cautious shifts (see, e.g., Isenberg 1986 for a survey). Recent studies from experimental economics also find a movement towards more extreme positions in group decisions, although somewhat more are in favor of the cautious shift (see e.g. Bateman and Munro 2005; Shupp and Williams 2008; Sutter 2009).

However, as Trautmann and Vieider (2012) point out in a survey on social influences on risk attitudes, “[g]roup decisions are complex and involve voting rules and dynamic decision processes that make it potentially difficult to identify effects on risk attitudes” (p. 588, see also Sutter 2009; Bolton et al. 2015 for similar arguments). Thus, another stream of the literature tries to isolate the effects of single aspects of social settings on decisions made individually. Our study follows this approach: first, we study if an individual’s decision is influenced by whether it is also payoff relevant for someone else so that other-regarding concerns may impact choices. Second, we study whether choices are influenced by communication. Lastly, we are interested in how these two aspects of a social setting interact. Different from previous work we also consider prudence and temperance.

Standard economic theory does not make predictions for the influence of the social setting on individual decisions. Thus, in the following we will formulate null hypotheses for our main statistical tests assuming that the decision maker is not influenced by the social setting in any way. With respect to each hypothesis we will also discuss the related literature on second-order risk aversion as well as potential explanations for an influence of the social setting.

Decision making in social settings is often characterized by some form of responsibility for the payoffs of others. For simplicity we focus on situations with payoff commonality, i.e. a decision has the same payoff consequences for the decision maker and the person he is responsible for.^3^ The first (null) hypothesis we will test in our experiment is the following:

### **Hypothesis 1**

An individual’s decisions are not influenced by whether his lottery choices are also payoff-relevant for someone else.

Contrary to this hypothesis individuals harboring other-regarding concerns might very well be influenced by responsibility. Yet, prior evidence is not unequivocal and results vary based on the experimental designs and preference elicitation methods employed. For instance, Sutter (2009) employs an experimental design that is based on the risk elicitation method by Gneezy and Potters (1997). In this task subjects decide on how much to invest into a lottery that yields a gain with a probability of one third or a loss otherwise. Sutter observes a risky shift: subjects take more risk (that means they invest more) when they are responsible for others’ payoffs compared to when decisions are made individually. In contrast, using the same task (but different design) Füllbrunn and Luhan (2015) observe a cautious shift. Applying the multiple price list method by Holt and Laury (2002) neither Bolton et al. (2015), Humphrey and Renner (2011) nor Eijkelenbloom and Vostroknutov (2016) find a significant influence of responsibility. In this method people decide repeatedly between two lotteries with constant positive outcomes but varying probabilities.

Furthermore, Bolton and Ockenfels (2010) and Andersson et al. (2016) compare choices between a safe positive payoff and a 50–50 lottery with and without responsibility. Both studies find no significant responsibility effect with respect to payoffs in the gain domain. In addition, Andersson et al. (2016) observe weakly significantly more risk taking under responsibility in the loss domain. Also considering 50–50 lotteries over gains and loss separately, Pahlke et al. (2015), observe a cautions shift with respect to the gain domain and no significant difference with respect to the loss domain. Furthermore, focusing only on the gain domain in another experiment, they observe no significant difference in lotteries with a winning probability of 10% and a cautious shift in lotteries with a winning probability of 90%.^4^

Despite the mixed evidence several arguments suggesting an influence of responsibility have been made. For example, Bolton et al. (2015) argue that responsibility for others might be equated with caution which would lead to more risk averse choices in general (see also Pahlke et al. 2015). Another explanation they present is the aim to avoid blame for bad outcomes (see also Eijkelenbloom and Vostroknutov 2016). A related explanation for shifts in choices is the idea that decision makers care about the preferences of others but have systematically biased estimates of these preferences as discussed by Bolton et al. (2015), Füllbrunn and Luhan (2015) and Pahlke et al. (2015).^5^ Also increased size of overall stakes in decisions with responsibility may lead to more cautious behavior: Once subjects take into account the payoff of others the stakes of their decisions increase (see also Pahlke et al. 2015; Vieider et al. 2016). It is well known that increasing the stakes of lotteries leads to more risk averse decisions (see e.g. Binswanger 1981; Holt and Laury 2002; Haering et al. 2017).^6^

Social settings often also offer the possibility to communicate with others before making decisions under uncertainty. Before considering the interaction of responsibility with communication, we focus on the pure effect of communication. The respective (null) hypothesis we test in our experiment is the following:

### **Hypothesis 2**

An individual’s decisions are not influenced by the preferences of someone else he can communicate with.

However, prior evidence from the experimental literature on second-order risk preferences suggests that people are influenced by knowing the preferences of others (even without responsibility). Most studies considering this setting do not focus on bidirectional communication but simply inform individuals about other people’s choices. These studies include the work by Yechiam et al. (2008), Cooper and Rege (2011), Lahno and Serra-Garcia (2015), Gioia (2017) and Bolton et al. (2015). Different from these studies Bougheas et al. (2013) allow participants to exchange free-form messages. However, all of the studies suggest that behavior between decision makers correlates once they can communicate or are informed about preferences of others.

Cooper and Rege (2011) present a systematic discussion of potential drivers of peer effects. One explanation is a fundamental taste for conforming to the decisions of others (see also Lahno and Serra-Garcia 2015). Another explanation they discuss is that imitating the choices of others functions as a heuristic applied to aid complex decision-making. Closely related is the idea of knowledge spillovers. People who can exchange information about the decision situation may learn from each other and make more similar decisions thereafter. Note that these explanations may apply with and without payoff commonality. Another explanation Cooper and Rege (2011) discuss is based on social regret: without payoff commonality people may choose like others to avoid a situation in which others made a different choice resulting in higher payoffs [for related explanations see also Lahno and Serra-Garcia (2015) on envy and Bolton et al. (2015) on the avoidance of ex-post inequality in payoffs].^7^

Lastly, we will analyze the combination of other-regarding concerns with communication. In this scenario we consider the situation in which individuals make decisions with responsibility for the payoffs of someone else and can communicate with the affected person. Thus we now consider whether responsibility changes the influence the communicating partner has on the decision maker. Accordingly, with respect for settings with communication the third (null) hypothesis that we test for is:

### **Hypothesis 3**

An individual’s decisions are influenced in the same way by someone for whom they are payoff-relevant as by someone for whom they are not payoff-relevant.

Prior evidence on the effect of communication with and without responsibility is limited. To our knowledge, only Bolton et al. (2015) compare both situations by also conducting a treatment with responsibility and information on peer behavior. They find that with and without responsibility subjects follow choices of peers. However, the effect is asymmetric: With responsibility they are more likely to follow those who are more risk averse than themselves than those who are less risk averse. Without responsibility decision makers are equally likely to adjust their decisions towards the preferences of peers.

Theoretical considerations may also lead us to expect a mediating effect of responsibility on peer effects. On the one hand the previously mentioned explanations for a responsibility effect may depend on the availability of a communication channel. For example, misaligned beliefs about others’ preferences may easily be corrected by communication. On the other hand, also explanations for a communication effect may depend on payoff commonality. Especially social regret should be absent in situations in which participants earn the same payoffs. An additional explanation for an influence of communication in settings with responsibility and payoff commonality is guilt aversion. As Charness and Dufwenberg (2006) summarize: “A guilt-averse player suffers from guilt to the extent he believes he hurts others relative to what they believe they will get. Therefore, he is motivated by his beliefs about others’ beliefs” (p. 1583). That means, a guilt-averse decision maker may be influenced by others who share information about their beliefs, e.g. what kind of lottery choice they expect him to make. In this case a guilt-averse decision maker may gain utility by fulfilling these expectations.

## Experimental design

### Lotteries

The aim of this study is to compare higher-order risk preferences of individuals in different social settings. Therefore, it is more informative to measure the intensity rather than the mere direction of these risk preferences. The only experiment measuring intensities so far is the study by Ebert and Wiesen (2014). They base their elicitation method on the risk apportioning lotteries introduced by Eeckhoudt and Schlesinger (2006) and on the compensation premia introduced by Crainich and Eeckhoudt (2008). The elicitation method of Ebert and Wiesen (2014) follows a multiple price list approach, as known from Cohen et al. (1987), Tversky and Kahneman (1992), or Holt and Laury (2002), among others. However, in contrast to common price list approaches in which probabilities are varied (while outcomes are held constant), Ebert and Wiesen (2014) vary outcomes (while holding probabilities constant). We apply their elicitation method in our experimental design.

**Fig. 1:**
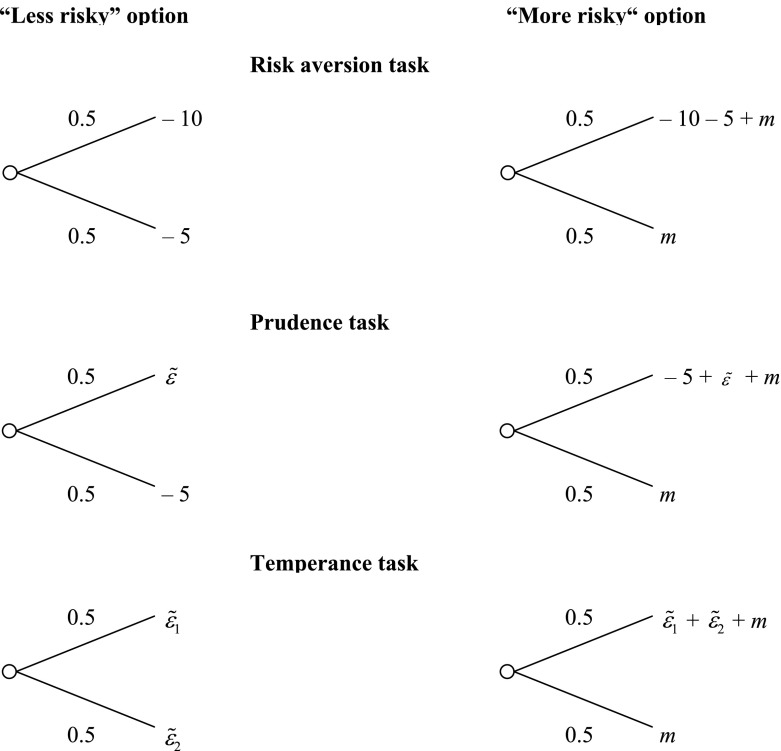
Lottery pairs for risk aversion, prudence, and temperance tasks. *Note* The different stage-dependent zero-mean risks are denoted by $\tilde{\varepsilon }$ and the potential compensation premia by *m*

Subjects face pairwise lotteries in three different tasks: a risk aversion task, a prudence task, and a temperance task, consisting of one, three, and two stages, respectively. In each stage, the subjects face 20 decision situations that are displayed by two lotteries, one “less risky” and one “more risky” (see Fig. 1).

Following Ebert and Wiesen (2014), the lotteries are displayed as draws from one or more urns and outcomes are framed as losses.^8^ However, each subject receives an endowment that depends on the task. In the risk aversion task the endowment is €25, while in the prudence and temperance tasks the endowments are €20 and €17.50, respectively (for each stage within a task). In the prudence and temperance tasks also zero-mean risks $\tilde{\varepsilon }$ matter. Depending on the stage, $\tilde{\varepsilon }$ takes the values $\tilde{\varepsilon } = [0.5,\,7;\,0.5,\, - 7]$, $\tilde{\varepsilon } = [0.8,\,3.5;\,0.2,\, - 14]$, and $\tilde{\varepsilon } = [0.8,\, - 3.5;\,0.2,\,14]$ in the prudence task.^9^ In the temperance task subjects face two zero-mean risks, $\tilde{\varepsilon }_{1}$ and $\tilde{\varepsilon }_{2}$. Depending on the stage, these risks take the values $\tilde{\varepsilon }_{1} = [0.5,\,7;\,0.5,\, - 7]$ and $\tilde{\varepsilon }_{2} = [0.5,\,3.5;\,0.5,\, - 3.5]$ or $\tilde{\varepsilon }_{1} = [0.8,\, - 2.8;\,0.2,\,11.1]$ and $\tilde{\varepsilon }_{2} = [0.8,\,2.8;\,0.2,\, - 11.1]$.

In each of the six stages the subjects face 20 decision situations in which the compensation premia *m* varies in steps of €0.25. We collect data using the ranges [−2.5, 2.25] and [−0.5, 4.25].^10^ The risk neutral compensation is €0.00, leading to an expected value of €17.5 for each stage. Choosing a compensation premium of less than zero reveals (2nd-, 3rd-, or 4th-order) risk loving behavior while choosing a compensation premium above zero reveals (2nd-, 3rd-, or 4th-order) risk averse behavior. An individual’s risk premium *m* is the smallest amount that makes him choose the “more risky” lottery.

### Treatments

The experiment is split into two parts. In Part I, we elicit the preferences for all subjects individually. This part follows Ebert and Wiesen (2014). For a more detailed description of Part I of the experiment please see “Online Appendix A” where we give an example on how to elicit higher-order risk preferences and “Online Appendix B” that shows the instructions that were given to the participants. The private elicitation of individual preferences serves as our Baseline treatment. This approach allows us to control for individual preferences when comparing behavior across treatments in Part II.

In Part II, we vary the social setting in a simple 2 × 2 design yielding the baseline treatment and three additional treatments. In each of these three additional treatments, the individual is paired with another subject, who is referred to as the partner. The role of the partner varies across treatments. Subjects are randomly distributed across the four treatments and two roles (individual and partner). Since some individuals face the Baseline treatment also in Part II, we are able to assess the effect of repeating the decision task (and control for it in our regression analyses). In the three additional treatments, the elicitation method follows the elicitation method of the Baseline treatment. That is, the second part is also organized in six stages, where the stages of the second part equal the stages of the first part. Note that we ran all four treatments within each session. This minimizes the potential influence of session-level effects on our results. “Online Appendix C” summarizes the order of events.

In the other-regarding concerns treatment O, individuals make decisions that not only determine their own payoff but also the payoff of the partner who does not make any payoff-related decisions. Both subjects receive the same payoff. Neither can communicate (the partner is passive) but through responsibility for the partner’s payoff other-regarding considerations might influence behavior.

In the communication treatment C, individuals can chat with their partner while both face the same decisions. These choices are only payoff-relevant for themselves individually. In this way, decisions can be influenced by communication but not by other-regarding concerns. We used anonymous free-form chat messages that subjects exchanged on a computer screen. This allows us to study the content of the messages while avoiding confounding effects from face-to-face interaction, such as those due to reputation.

Finally, in the CO treatment, individuals make decisions that determine their own payoff and also the payoff of the partnering subject. Again, both subjects receive the same payoff. However, in this treatment they can also chat with the otherwise passive partner, allowing for communication and other-regarding concerns to influence decisions. For a more detailed description of Part II of the experiment please see the instructions that were given to the participants shown in “Online Appendix D”.

Table 1 summarizes the four treatments. Within each treatment we also vary the range of the price list’s grid. Following Ebert and Wiesen (2014), we refer to the range of [–0.5, 4.25] as Shift and to the range of [–2.5, 2.25] as no shift. In 12 of the 15 sessions, 21 subjects participated in each session, 3 of them in the Baseline treatment and 6 of them in each of the remaining treatments. In the remaining 3 sessions, 22 subjects participated in each session. In these sessions, we collected one additional observation for the Baseline treatment. We had to exclude six subjects (three pairs) that participated in the CO treatment of the first session due to a display error. Eight sessions were conducted with the shift grid and seven sessions with the no shift grid, yielding the number of participants (*N*) displayed in Table 1.

**Table 1:** Treatments

Treatment	Part 1		Part 2		*N* (shift/no shift)
	Communication	Other-regarding concerns	Communication	Other-regarding concerns	
Baseline	No	No	No	No	48 (24/24)
O	No	No	No	Yes	90 (48/42)
C	No	No	Yes	No	90 (48/42)
CO	No	No	Yes	Yes	84 (42/42)

### Experimental procedure

The experiment was conducted at the Essen Laboratory for Experimental Economics (elfe) at the University of Duisburg-Essen, Germany. Altogether, 312 subjects participated and sessions lasted 90 min each. We only consider the behavior of same-gender pairs. Given that several studies have suggested that women’s behavior is more risk averse than men’s (for surveys, see Eckel and Grossman 2008; Croson and Gneezy 2009), the gender make up of the participants could influence behavior in social settings. The results on higher-order risk preferences show that women are—with weak significance—also more prudent and temperate than men (Ebert and Wiesen 2014). Due to our focus on individual decision making, interactions between participants of different gender would have required several additional treatments. We chose to study male subjects instead of females only for recruitment reasons: there are more males in the subject pool. Subjects were recruited using the software ORSEE (Greiner 2004). The experiment was programmed with z-Tree (Fischbacher 2007), while communication between individuals and their partners was facilitated by the EasyChat software.^11^

Subjects entered the laboratory one after another and were randomly allocated to a workspace (by drawing a ball with their work space number from an urn) where they found the instructions for Part I. In this way, subjects were also allocated to the different treatments of Part II also allowing us to control the number of subjects in each treatment. Questions were answered in private at the subject’s workspace by the experimenter. At this time, communication between the subjects was not permitted. Part I started only after the subjects had successfully answered two comprehension questions about the instructions on their computer. These questions asked the subjects to calculate the payoffs of potential lottery outcomes in two decision situations.

In Part I the subjects first made their decisions in the risk aversion task. Subsequently, they made decisions in the prudence task with three stages and in the temperance task with two stages. Ebert and Wiesen (2014) do not find evidence for order effects when varying the order of the tasks. Therefore, we only ran one order of tasks. We started with the elicitation of risk aversion because the respective lotteries are somewhat simpler than the prudence and temperance lotteries that follow. We think that this makes it easier for the subjects to get used to the decision environment (see Noussair et al. 2014 for a similar reasoning). Note that this is also an argument for eliciting individual preferences in Part I before assigning subjects to the more complex treatments C, O and CO in Part II. We control for learning effects between Part I and Part II in our regressions (see the next section). The order of the stages within the prudence or the temperance task was randomly determined for each subject. After finishing all 120 decisions of the first part of the experiment, the subjects entered one of the four treatments of Part II and received the respective instructions for another 120 decisions. In the last step, the subjects’ payoff was determined randomly. To avoid wealth and averaging effects, we used the random payment technique (see e.g. Cubitt et al. 1998; Cox et al. 2015 for a discussion of this method). Therefore, only one of the 240 decisions was paid out after it was randomly chosen for each subject at the end of the experiment. Subjects earned €18.09 on average (minimum €1.00, maximum €34.00). Finally, the subjects were paid in private and left the laboratory one after another.

## Results

### Individual higher-order risk preferences

The results from Part I of our experiment can be directly compared to the findings presented by Ebert and Wiesen (2014). We follow their procedures in our data analysis and exclude subjects that switched more than two times in more than one of the six stages. Therefore, 35 out of 312 subjects (11%) are excluded, which is a higher percentage than the 6% reported by Ebert and Wiesen (2014) but is similar to studies using a multiple price list format [cf. 13% in Holt and Laury (2002) and 12% in Krieger and Mayrhofer (2012)]. For the analysis of Part I, we consider all of the subjects independent of the role that they will take in Part II (i.e., we consider individuals and partners).

We calculate an individual *i*’s risk premium in Part I $\bar{m}_{i}^{I}$ as the average $m_{ij}^{I}$ selected over the stages *j* within the respective task. The average risk premium is €1.17 in the risk aversion task, €1.53 in the prudence task, and €0.99 in the temperance task. All of the premia are significantly different from zero, which is the neutral switch point (*p* < 0.001, two-sided Wilcoxon signed-rank tests), revealing a tendency towards risk averse, prudent, and temperate behavior. Furthermore, the average risk premium differs significantly between tasks: it is largest in the prudence task and smallest in the temperance task (*p* ≤ 0.025). In their sessions with the same grid size for potential risk premia (“grid 0.25” in their terminology), Ebert and Wiesen (2014) observe slightly larger risk premia in the risk aversion task (€1.26) and the prudence task (€1.56), and a somewhat lower premium in the temperance task (€0.90).^12^

Overall, 17% of our subjects are risk loving $(\bar{m}_{i}^{I} < 0)$, 13% are risk neutral $(\bar{m}_{i}^{I} = 0)$, and 70% are risk averse $(\bar{m}_{i}^{I} > 0)$. Regarding prudence, we find that 6% of our subjects in Part I are imprudent, 4% are prudent-neutral, and 90% of our subjects are prudent. In the temperance task, 16% of the subjects are intemperate, 8% are temperate-neutral, and 76% are temperate. Our results are very similar to the findings by Ebert and Wiesen (2014). In their experiment, 66% of subjects are risk averse, 88% are prudent, and 75% are temperate.

**Fig. 2:**
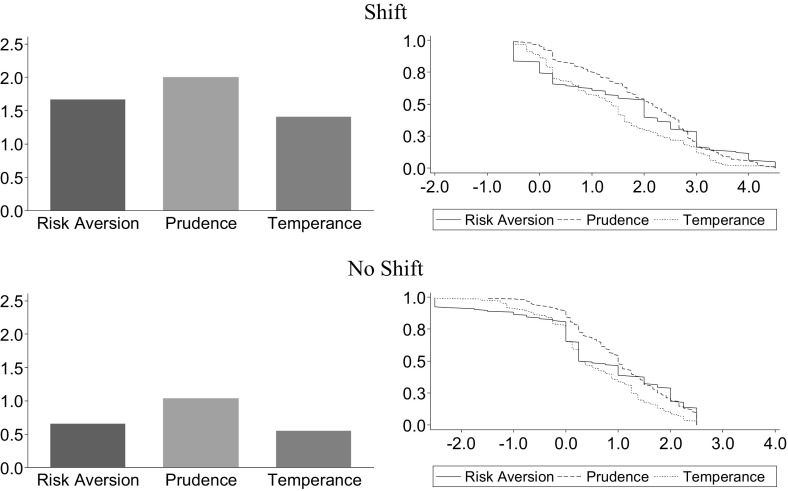
Means and cumulative distributions of risk premia in Part I

In addition, we observe that shifting the grid influences choices significantly. Like Ebert and Wiesen (2014), we find more risk averse, prudent and temperate behavior when the range of options is [−0.5, 4.25] in the Shift sessions than when it is [−2.5, 2.25] in the No Shift sessions (*p* < 0.001, two-sided Mann–Whitney-*U* tests). This observation may be due to the subjects’ well-known tendency to choose options in the middle of the price list (Ebert and Wiesen 2014; Abdellaoui et al. 2011). The left-hand side of Fig. 2 shows the average risk premia elicited with both types of grids. It reveals that the average risk premia are significantly higher for prudence than for risk aversion and temperance in the Shift sessions (*p* ≤ 0.044, two-sided Wilcoxon signed-rank tests) as well as in the No Shift sessions (*p* ≤ 0.004). The right-hand side of Fig. 2 shows the cumulative distributions of the risk premia. For example, the number of risk averse individuals reduces from 74% in the Shift sessions to 65% in the No Shift sessions. The number of participants classified as prudent and temperate reduces from 96 and 87% to 85 and 65%.

The large data set of individual decisions collected in Part I also allows for the identification of behavioral types. Deck and Schlesinger (2014) observe that decisions under uncertainty can be explained surprisingly well by mixed risk averse (Brocket and Golden 1987; Caballé and Pomansky 1996) or mixed risk loving preferences (Crainich et al. 2013; Ebert 2013). While mixed risk averters are risk averse, prudent, and temperate, mixed risk loving individuals are risk loving, prudent, and intemperate. Therefore, both mixed risk averse as well as mixed risk loving individuals are prudent.

On aggregate, we observe a higher risk premium in the prudence tasks than in the risk aversion and temperance tasks. This pattern is consistent with mixed risk aversion and mixed risk loving behavior. Following Deck and Schlesinger (2014), we also consider the individual choices of risk averters and risk lovers separately in the prudence and the temperance tasks. If people are either mixed risk averse or mixed risk loving, then the sign of their risk premia should coincide with respect to prudence but differ with respect to temperance. Of the 194 subjects who are risk averse, 94.3% demand a positive average premium in the prudence tasks and 82.0% do so in the temperance tasks. Of the 48 subjects who are risk loving, 83.3% demand a positive average premium in the prudence tasks but only 54.2% of them do so in the temperance tasks.^13^

### Higher-order risk preferences in social settings

#### Choices of decision-making individuals

In the following analysis of the influence of the social setting we ignore the partners but focus on the decision making individuals. Applying the same consistency check in Part II led to the exclusion of one additional subject. Analogous to Part I, the average risk premia $\bar{m}_{i}^{II}$ in all four treatments in Part II are significantly different from zero (*p* ≤ 0.001, two-sided Wilcoxon signed-rank tests). Thus, our experiment reveals that Ebert and Wiesen’s (2014) main finding on individual decisions that are made privately with respect to one’s own payoff applies more broadly. Based on the average risk premia, the majority of subjects are also classified as risk averse, prudent, and temperate in settings that allow for communication and other-regarding concerns (see Table 2). We summarize these observations as follows:

**Table 2:** Subject classification by treatment in Part II

	Risk averse (%)	Prudent (%)	Temperate (%)
Baseline	67	95	79
Treatment C	73	82	67
Treatment O	60	88	67
Treatment CO	80	88	80

##### **Observation 1**

Across all social settings we find that the behavior of the majority of the subjects is risk averse, prudent, and temperate.

Table 2 shows that, as in Part I, in Part II more choices are prudent than risk averse or temperate. This basic pattern is the same across all treatments. In the Baseline treatment the average risk premium is higher for prudence than for risk aversion and for temperance (*p* = 0.001, two-sided Mann–Whitney-*U* test) but it does not differ between temperance and risk aversion (*p* = 0.911). In treatment O the risk premium for risk aversion is weakly significantly smaller than for prudence (*p* = 0.087) and significantly larger than for temperance (*p* < 0.001). The risk premia for risk aversion and temperance only differ slightly (*p* = 0.101). The risk premia in the C and in the CO treatment for risk aversion increase relative to those for prudence. In both treatments, they do not differ significantly from each other (*p* ≥ 0.345). However, the risk premia for prudence are still significantly larger than for temperance (*p* ≤ 0.044). In treatment C the premium for risk aversion is weakly significantly larger than for temperance (*p* = 0.062). This difference is significant in the CO treatment (*p* = 0.004).

Our data set is not rich enough to analyze mixed risk averse and mixed risk loving patterns, or their interaction within treatments. But aggregated decisions in the Baseline treatment and, to a lesser degree, in the O treatment are in line with the concepts of mixed risk averse and mixed risk loving behavior, as observed by Deck and Schlesinger (2014): premia are higher for prudence than for risk aversion and temperance. The two communication treatments C and CO exhibit a different pattern: premia for prudence do not differ significantly from those for risk aversion. This might be driven by social interaction, which we will explore further in the following section.

#### Influence of partners on the decision-making individuals

Our experiment allows for quantification of the change of individual risk preferences associated with social settings. To identify the influence of social settings on risk attitudes, we run panel regressions with the risk premium as the dependent variable. Our data is censored: depending on the shift of the price list, roughly half of our subjects faced a price list ranging from −2.5 to 2.25 while the other half faced a price lift ranging from −0.5 to 4.25. Accounting for individual differences in censoring, we run a random effects interval regression estimated via maximum likelihood. We estimate an individual *i*’s risk premium *m*_*i*_^*k*^ within part *k* as

$$\begin{aligned} m_{i}^{k} = \beta_{0} + \beta_{1} \cdot m_{ - i}^{I} + \beta_{2} \cdot c_{i}^{k} + \beta_{3} \cdot c_{i}^{k} \cdot m_{ - i}^{I} + \beta_{4} \cdot o_{i}^{k} + \beta_{5} \cdot o_{i}^{k} \cdot m_{ - i}^{I} \hfill \\ + \beta_{6} \cdot co_{i}^{k} + \beta_{7} \cdot co_{i}^{k} \cdot m_{ - i}^{I} + \beta_{8} \cdot r^{k} + \beta_{9} \cdot s_{i} + \eta_{i} + \varepsilon_{i}^{k} . \hfill \\ \end{aligned}$$

We run one regression for risk aversion, one for prudence, and one for temperance. Within these regressions we do not consider average risk premia as dependent variable but choices within each stage *j* of Parts I and II. We drop the index *j* to simplify notation. Per subject, this yields two observations from the stages of the risk aversion task, six observations from the stages of the prudence task, and four observations from the stages in the temperance task—one half from Part I, the other from Part II. This estimation approach allows us to assess the influence of potential communication and other-regarding concerns on behavior while controlling for individual differences in risk attitudes (via the observations in Part I) and any potential repetition effects (via the Baseline treatment).

The potential influence of communication and other-regarding concerns across treatments are indicated by the binary variables *c*, *o* and *co*. The variable *c* takes the value of one if subjects can communicate (in treatments C and CO) while *o* takes the value of one if subjects are responsible for a partner so that other regarding concerns may influence behavior (in treatments O and CO). Finally, *co* equals one if both are the case; that is, this dummy captures any complementary effects of communication and other-regarding concerns. These dummies are also interacted with the partner’s risk premium in Part I $m_{ - i}$. The interaction terms capture the varying influences the partner has on the decision maker. The repetition of a stage in Part II is indicated by the dummy *r*, which captures potential learning effects across parts. The shift of the grid of risk premia from [−2.5, 2.25] to [−0.5, 4.25] is indicated by the dummy *s*. Furthermore, we assume independent and normally distributed error terms denoted by *η* and *ε* that capture differences between individuals and idiosyncratic effects within stages.^14^

The resulting average marginal effects and standard errors are presented in Table 3, while Table 4 contains explanations of the independent variables. In model (1) we only consider the aggregate treatment effects and do not consider the different influences that the partners may have on the decision making individuals. In model (2) we also take into account this potential influence. In both models and across tasks, the results reveal a consistent positive effect of shifting the grid but no systematic learning effect across parts.

**Table 3:** Random effects interval regressions on risk premia

	(1)			(2)		
	Risk aversion	Prudence	Temperance	Risk aversion	Prudence	Temperance
$m_{ - i}^{I}$	0.066	0.040	0.044	−0.037	0.030	0.023
Partner’s influence	(0.075)	(0.040)	(0.049)	(0.094)	(0.052)	(0.063)
*c*	0.120	−0.181	0.017	0.073	−0.219	−0.093
Dummy: treatments with communication C and CO	(0.213)	(0.151)	(0.167)	(0.270)	(0.198)	(0.184)
*c·m*_−*i*_^*I*^				0.037	0.031	0.144
Interaction: partner’s influence in other-regarding concerns treatments C and CO				(0.132)	(0.085)	(0.095)
*o*	−0.115	−0.272^*^	0.013	−0.240	−0.092	0.075
Dummy: treatments with other-regarding concerns O and CO	(0.203)	(0.143)	(0.158)	(0.231)	(0.181)	(0.170)
*o*·*m*_−*i*_^*I*^				0.125	−0.119	−0.099
Interaction: partner’s influence in other-regarding concerns treatments O and CO				(0.110)	(0.075)	(0.089)
*co*	0.497^**^	−0.140	−0.140	0.149	−0.722^***^	−0.225
Dummy: treatment with other-regarding concerns and communication CO	(0.253)	(0.173)	(0.194)	(0.294)	(0.242)	(0.226)
*co*·*m*_−*i*_^*I*^				0.295^**^	0.363^***^	0.085
Interaction: partner’s additional influence through other-regarding concerns and communication in CO				(0.136)	(0.107)	(0.115)
*r*	−0.062	0.105	−0.082	−0.061	0.099	−0.085
Dummy: decisions in Part II	(0.152)	(0.106)	(0.117)	(0.149)	(0.105)	(0.117)
*s*	0.759^***^	0.600^***^	0.577^***^	0.734^***^	0.605^***^	0.569^***^
Dummy: decisions with shifted grid	(0.246)	(0.185)	(0.198)	(0.248)	(0.185)	(0.197)
*N*	296	888	592	296	888	592
*Log*-*likelihood*	−503.853	−1435.250	−966.830	−499.980	−1428.333	−964.890

Reports average marginal effects of the respective coefficients on observed risk premia

Standard errors are given in parentheses

^*^ *p* < 0.10, ^**^ *p* < 0.05, ^***^ *p* < 0. 0 1

In model (1) the regression results do not suggest any influence of the social setting on temperance: Neither the communication dummy *c* nor the other-regarding concerns dummy *o* indicates a significant difference to the Baseline treatment, in which decisions are made privately and are only payoff-relevant for the decision maker. Also, there appears to be no complementary effect of communication and other-regarding concerns as the *co* dummy is insignificant as well. With respect to risk aversion and prudence, however, the social setting matters: the regressions reveal a significant positive effect of other-regarding concerns in combination with communication (*co*) on the premium in the risk aversion task. The regressions also reveal a weakly significant negative effect of other-regarding concerns (*o*) on the premium in the prudence task. Our (null) hypothesis 1 states that an individual’s decisions are not influenced by whether they are also payoff relevant for someone else (treatment O). Based on the latter finding we can only reject (null) hypothesis 1 for prudence (with weak significance):

##### **Observation 2**

We find no differences in risk aversion and temperance but less prudence when an individual’s decisions are also payoff-relevant for someone else.

In model (2) we focus on the size of the influence the partner has on the decision maker. As expected, the partner does not have a significant influence in either the Baseline treatment or in the O treatment. Because no communication takes place, no influence is possible. This might be different in the communication treatments since subjects are allowed to exchange information on each other’s preferences and thus might adjust their choices accordingly. Our (null) hypothesis 2 states that there is no influence of the partner, even in the treatments with communication. Therefore, we test whether the correlation between preferences differs from that in the Baseline treatment. These differences would be captured by the *c*·*m*_−*i*_ interaction term. We find that the coefficients are positive but not significant at reasonable levels regarding risk aversion, prudence, or temperance. Thus, we cannot reject (null) hypothesis 2:

##### **Observation 3**

We do not find a significant influence of the partner on the individual’s decisions when the partner can communicate his preferences to the decision maker. This observation is made with respect to risk aversion, prudence, and temperance.

Lastly, we study whether communication matters more in combination with other-regarding concerns, e.g. through responsibility for the partner’s payoff. Our (null) hypothesis 3 states that we should find no additional effect of the partner’s preferences on the decisions in the CO treatment. This additional effect would be captured by the interaction term *co*·*m*_−*i*_ in model (2). Table 3 shows that this coefficient is not significant with respect to temperance but it is significantly positive with respect to risk aversion and prudence. Say a decision maker is paired with a partner who demands a €1 higher risk premium. In the risk aversion stage he will demand €0.30 more for himself in CO than in the other treatments on average. In the prudence stage, he will demand €0.36 more. Thus, our results not only suggest that the social dimension is important for risk aversion and prudence but they also suggest that communication mainly matters in combination with other-regarding concerns. Therefore, we reject hypothesis 3 with respect to risk aversion and prudence:

##### **Observation 4**

We find a significantly stronger influence of the partner on the individual’s decisions when they are also payoff relevant for the partner. This observation is made with respect to risk aversion and prudence but not temperance.

The results of model (2) also suggest that the more risk averse behavior in CO found in model (1) appears to be driven by the matching of decision makers with risk averse partners: In model (2) the *co* dummy turns insignificant after controlling for the influence of the partner on the decision maker. To shed more light on the peer effects in treatments C and CO, we present a breakdown of choices in Table 4 (also see “Online Appendix E” for figures illustrating the individual choice patterns across treatments). We summarize the relative frequencies of people who make more, the same number of, or less risk averse choices after having the chance to communicate with the partner. For both treatments we split subjects into two groups: those who are matched with someone less risk averse ($\bar{m}_{ - i}^{I} < \bar{m}_{i}^{I}$) are shown on the left-hand side and those matched with someone more risk averse ($\bar{m}_{ - i}^{I} > \bar{m}_{i}^{I}$) on the right-hand side. Separate Fisher’s exact tests for risk aversion, prudence and temperance indicate that distributions differ between both groups in treatment CO (*p* ≤ 0.008, Fisher’s exact tests) but not in treatment C (*p* ≥ 0.181). Thus, in line with the regression results for risk aversion and prudence (but not temperance) we find a significant influence of the partner in CO but not in C. Furthermore, we analyze the communication content of the No Shift sessions. We find that this relationship is also mirrored in the communication patterns: Even though the frequency of mentioning preferred choices does not differ between treatments, in CO agreement with the other person’s choice is more often voiced than in C (see “Online Appendix F” for details). It is important to note that the partner’s influence sometimes leads to a shift in preferences. For example, of all risk-averters ($\bar{m}_{i}^{I} > 0$) in CO 22% of those matched with a non-risk-averter did not choose in a risk-averse manner in Part II but made risk neutral or risk-seeking choices ($\bar{m}_{i}^{II} \le 0$).

**Table 4:** Changes in risk premia in C and CO depending on partner’s preferences

		Treatment C		Treatment CO	
		$\bar{m}_{ - i}^{I} < \bar{m}_{i}^{I}$ (%)	$\bar{m}_{ - i}^{I} > \bar{m}_{i}^{I}$ (%)	$\bar{m}_{ - i}^{I} < \bar{m}_{i}^{I}$ (%)	$\bar{m}_{ - i}^{I} > \bar{m}_{i}^{I}$ (%)
Risk aversion	$\bar{m}_{i}^{II} > \bar{m}_{i}^{I}$	23	47	13	65
	$\bar{m}_{i}^{II} = \bar{m}_{i}^{I}$	46	40	33	20
	$\bar{m}_{i}^{II} < \bar{m}_{i}^{I}$	31	13	53	15
Prudence	$\bar{m}_{i}^{II} > \bar{m}_{i}^{I}$	27	47	14	55
	$\bar{m}_{i}^{II} = \bar{m}_{i}^{I}$	0	13	7	20
	$\bar{m}_{i}^{II} < \bar{m}_{i}^{I}$	73	40	79	25
Temperance	$\bar{m}_{i}^{II} > \bar{m}_{i}^{I}$	19	36	6	55
	$\bar{m}_{i}^{II} = \bar{m}_{i}^{I}$	25	36	12	20
	$\bar{m}_{i}^{II} < \bar{m}_{i}^{I}$	56	27	82	25

Bolton et al. (2015) observe that the influence of peers is asymmetric in the sense that decision makers are more likely to adjust decisions toward more risk averse choices in settings with information about others’ preferences and responsibility. However, we find no significant asymmetry after calculating the additive inverse of the distribution of one of the groups and comparing it to the original distribution of the other group. We run separate Fisher’s exact tests for risk, aversion and prudence in both treatments. All of them turn out insignificant (*p* ≥ 0.203).

## Discussion and conclusion

Previous experiments on higher-order risk preferences have focused on eliciting individual decisions that are made in isolation and only affect the decision maker’s payoff. In this paper, we extend this line of research by studying the social dimension of higher-order risk preferences. We believe this aspect of higher-order risk preferences is of particular importance because many risky decisions are made in social settings. Couples buying a house or board members considering building a plant will take into account background risks when making their decisions. However, none of them will decide in isolation and their choice affects the payoff of more than one person. This study builds on the study by Ebert and Wiesen (2014), who introduced a method for eliciting the strength of higher-order risk preferences. We apply their method and systematically vary how communication and other-regarding concerns can influence decisions.

We find that the majority of subjects is risk averse, prudent, and temperate across social settings. In addition, our findings on decisions made individually as well as with responsibility for the payoff of others are in line with recent findings on mixed risk averse and mixed risk loving preferences by Deck and Schlesinger (2014). In these treatments risk premia are higher for prudence than for risk aversion and temperance.

Moreover, other regarding concerns do not impact preferences regarding risk aversion and temperance when individuals cannot communicate. However, we observe somewhat less prudent choices when subjects are also responsible for someone else’s payoff. With respect to risk aversion our null finding is in line with previous results. Studies also estimating risk preferences using 50–50 lotteries in the loss domain either find weakly significantly more risk taking with responsibility (Andersson et al. 2016) or no significant differences (Pahlke et al. 2015). It has been argued that decision makers may harbor other-regarding concerns in these settings but have systematically biased estimates of the preferences of others (Bolton et al. 2015; Füllbrunn and Luhan 2015; Pahlke et al. 2015; Eriksen and Kvaloy 2010; Pollmann et al. 2014). A similar bias could explain the change in prudence we observe. However, surprisingly there appears to be no such discrepancy with respect to risk aversion or temperance.

We do not find a significant influence of partners on decision-making individuals when they are able to communicate but only the individual himself is affected by the decision. With respect to risk aversion this result is at odds with several studies finding peer effects in risk taking. In most of these studies decision makers are automatically informed about the preferences of others which could give rise to experimenter demand effects. However, please note that Bougheas et al. (2013) find peer effects also with free-form communication.

Lastly, we observe that decision-making individuals are influenced significantly more strongly by the preferences of a partner when they are able to communicate and choices are payoff-relevant for both of them. Our regressions reveal that this finding applies to risk aversion and prudence but not to temperance. With respect to risk aversion similar findings have been obtained by Bolton et al. (2015). Due to the commonality of payoffs this peer effect cannot be driven by social regret, i.e. by decision makers trying to avoid payoff differences in lottery outcomes (Cooper and Rege 2011; Lahno and Serra-Garcia 2015; Bolton et al. 2015). Our analysis of chat content reveals that in the treatment with payoff commonality subjects mention agreement with the other’s choice more often than without payoff commonality. This suggests that reaching consensus may drive some of the peer effects we observe. One explanation is that decision-making individuals harbor other-regarding concerns and adjust their choices according to the preferences of the partners. Another explanation is based on guilt aversion. It proposes that decision-making individuals like to fulfill the expectations of their partners. However, more research is needed to disentangle potential explanations for peer effects.

## Electronic supplementary material

Below is the link to the electronic supplementary material.

Supplementary material 1 (DOCX 365 kb)

## Footnotes

1 As Gollier and Pratt (1996, p.1114) point out: “any unfair risk can be expressed as the combination of a mean-zero risk and a deterministic downward shift in wealth”.

2 An exception is the very recent work by Ebert and van de Kuilen (2015). They study—among other things—situations involving outcomes accruing to another person. However, in their study the decision-maker decides over the other person’s wealth only. Therefore their results cannot be compared directly to ours.

3 Please note that for this reason we do not focus on theories of social preferences such as reciprocity or inequity aversion (Fehr and Schmidt 1999; Bolton and Ockenfels 2000).

4 Several studies also compare decisions that are payoff-relevant to the decision maker to decisions that are only payoff-relevant for someone else (and not to the decision maker). For example, Füllbrunn and Luhan (2015) and Eriksen and Kvaloy (2010) observe a cautions shift based on the task by Gneezy and Potters (1997). Using the same task Pollmann et al. (2014) observe more risky decisions for others instead. However, in their study the gap disappears after repetition. Applying 50–50 lotteries, Andersson et al. (2016) do not observe differences in the gain domain but weakly significantly more risky choices for others in the loss domain. See also the survey by Trautmann and Vieider (2012).

5 See also Eriksen and Kvaloy (2010) and Pollmann et al. (2014) who mention the potential discrepancy between estimated and actual preferences with respect to decisions that are only payoff-relevant for someone else.

6 Pahlke et al. (2015) conclude that their findings are at odds with a stake size explanation as well as a carefulness norm explanation. Instead they argue that responsibility may amplify the behavioral pattern captured by prospect theory. In a follow-up study, Vieider et al. (2016) confirm this conjecture by structurally estimating a prospect theory model.

7 Note that these drivers per se only explain aggregate differences in the variance of risk preferences but not differences in average risk preferences. Different average risk preferences can be explained if these drivers are asymmetric or correlate with an individual’s risk preference. For example, if only risk averse people imitate others or if only more risk averse choices are worth imitating then learning about other’s risk preferences will also yield less risk averse decisions on aggregate. Of the studies considering a potential effect of communication on the level of risk aversion, Yechiam et al. (2008) observe more risky decisions once individuals are informed about the choices of others while Bougheas et al. (2013), Bolton et al. (2015), Lahno and Serra-Garcia (2015) and Gioia (2017) observe no significant effect on the level of risk taking.

8 Note that Deck and Schlesinger (2010) and Maier and Rüger (2012) find no evidence of a loss framing on the prevalence of higher-order risk preferences. Still, framing might influence the strength of higher-order risk preferences and the impact of the social setting. Also, the prudence and temperance lotteries are displayed in compound form as separate draws from two or three urns which may also influence the measurement of higher-order risk preferences (Haering et al. 2017; Deck and Schlesinger 2016).

9 Note that the background risk in the second and third stage of the prudence task is skewed. A left-skewed (right-skewed) background risk decreases (increases) the difference in kurtosis of the overall lotteries, which might also be important with regard to temperance (see e.g. Mayrhofer 2017). See Ebert and Wiesen (2014) for the statistical features of the lottery pairs (“web appendix, section B”).

10 Ebert and Wiesen (2014) vary the range of the risk premium in the same way. In addition, they vary its grid size (€0.25 and €0.50). They observe that the range has an influence on choices while grid size has not. Because we select a grid size of €0.25, our data corresponds to their “fine grid” data.

11 See www.ec3hp.de for more information. Unnecessary components of EasyChat’s user interface were hidden using Zorro, see www.gabrieleponti.com/software.

12 Like Ebert and Wiesen (2014), we also observe positive and significant correlations between the risk premia as measured by the Spearman rank correlation coefficients (*p* < 0.001): the risk premium in the risk aversion task is positively correlated with the premium in the prudence task (*r*_*s*_ = 0.468) as well as with the premium in the temperance task (*r*_*s*_ = 0.349). The premia in the prudence and in the temperance task are also positively correlated with each other (*r*_*s*_ = 0.482).

13 Note, however, that in the Shift sessions only 2 of the 20 available premia of the decision grid to choose from were negative. This could induce the subjects to choose larger premia overall. In fact, we find that risk lovers in the Shift sessions ask for positive premia that differ significantly from zero in the prudence as well as in the temperance tasks (*p* ≤ 0.003, two-sided Wilcoxon signed-rank tests). In the No Shift sessions, risk loving subjects only ask for significantly positive risk premia in the prudence tasks (*p* = 0.011) but not in the temperance tasks (*p* = 0.957). In the Shift as well as in the No Shift sessions, risk averters ask for positive premia that differ significantly from zero in the prudence and the temperance tasks (*p* < 0.001).

14 To estimate meaningful effects of the respective partners on the decision making individuals, we place additional restrictions on the data. First, we only consider individuals who are consistent in Part I *and* Part II. With respect to the C and CO treatments, we only consider pairs in which both subjects decided consistently in Part I. This leads to the exclusion of another 18 subjects. Because of potential correlation within the symmetric pairs of the C treatment, we only consider one randomly selected decision maker of each pair.
