# Supplementary material for: Higher-order risk preferences in social settings
Source: Exp Econ. 2017 Sep 8;21(2):434–56. doi: 10.1007/s10683-017-9541-4 (PMC5913393; doi:10.1007/s10683-017-9541-4)
Supplement: Supplementary file 1 — Supplementary material 1 (DOCX 365 kb) [file 10683_2017_9541_MOESM1_ESM.docx]

**Online Appendix**

1. **Eliciting higher-order risk preferences**

Supplemental Figure 1 gives an example of the elicitation of (higher-order) risk preferences. It shows the stage of the prudence task with $\tilde{\varepsilon}$ = [0.8, 3.5; 0.2, -14] and includes 20 decision situations.

In each decision situation the subjects state which of the two risky events, one more and one less risky, they prefer (in Supplemental Figure 1: Option A or Option B,). Both risky events comprise a random draw that is depicted as an urn with two balls named Up and Down, where both balls can be drawn with equal probability.

We will start with Option B on the right-hand side of Supplemental Figure 1: If the ball Up is drawn, then the subject faces a second random draw which is also shown as an urn. In the second random draw, the subject receives € 3.5 with a probability of 80% and loses € 14 with a probability of 20% (the urn contains 8 balls with a payoff of € 3.5 and 2 balls with a payoff of € -14). If the ball Down is drawn, then the subject loses a fixed amount of € 5 from his endowment of € 20.

In Option A: If the ball Up is drawn, then the subject loses € 5 and faces the second random draw. Since option A is the risky event, a compensation amount is added to both situations Up and Down displayed within the blue box. The amount depends on the decision situation, which is selected on the right-hand side of the screen in Supplemental Figure 1. In this example, the amount takes a value of € 3. The subject now has to decide which of the two options he prefers for each of the 20 amounts shown on the right-hand side. However, the subjects do not need to stick to a certain order of their decisions. After finishing all 20 decisions, a button labeled next appears and the subjects can leave the stage by clicking on it. However, before the next stage starts the subjects have to confirm their decisions.


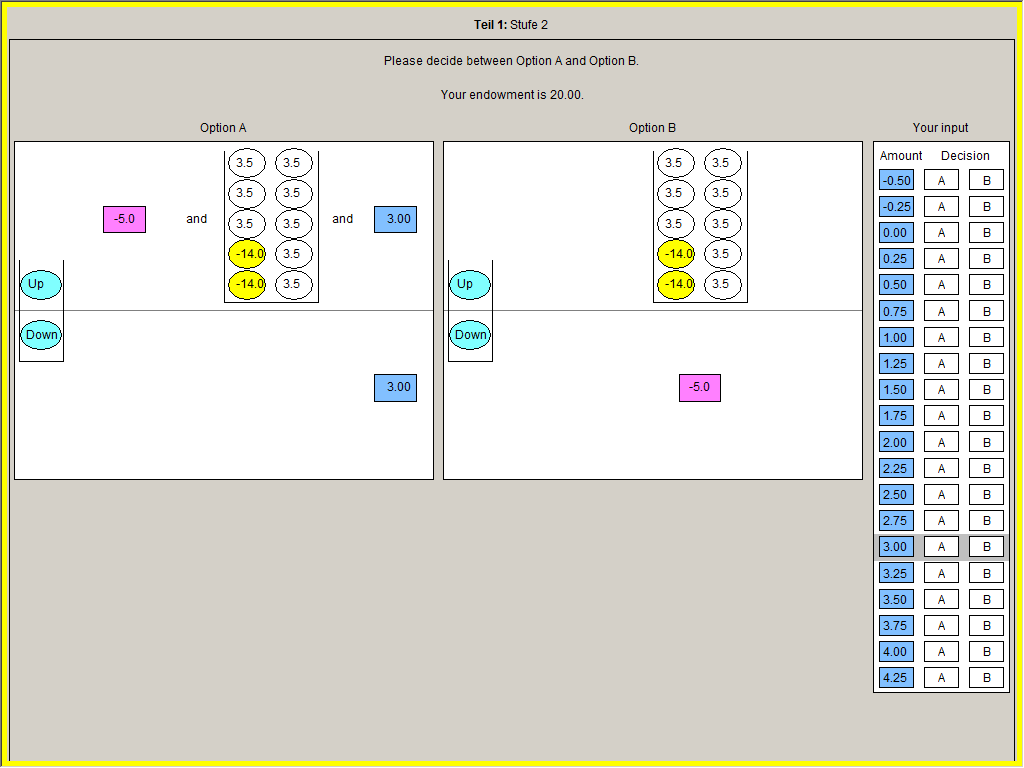


**Figure A1: Screenshot of a decision screen in the prudence task**

1. **Instructions Part I**

[*Translated from German; Instructions of Part I follow Ebert and Wiesen (2014)*]

**General Information**

In the following experiment, you will make several decisions. By following the instructions you can earn money. Your earnings depend on your choices and on chance. It is therefore very important that you read the instructions carefully. Decisions will be made using a computer. During the experiment you are not allowed to talk to the other participants. Whenever you have a question, please raise your hand or open your cabin door. The experimenter will answer your question in private at your place. If you disregard these rules, you can be excluded from the experiment. Then you will receive no payment.

In the experiment **all amounts are stated in** **Euro**. At the end of the experiment, you will be paid your earnings in cash.

The experiment consists of two different parts. Before each part starts, you will receive detailed instructions. Please notice that neither your decisions from the first part of the experiment nor your decisions from the second part of the experiment will influence the other part of the experiment.

**At the end of the experiment one of the two parts is chosen randomly for your payoff. The drawing of one of the both parts occurs with equal probability.**

**Structure of the experiment**

This part of the experiment is divided into six stages. Note that all stages are equally relevant for your payoff. The six stages comprise decision problems where risky events play a role. In each of the decision problems, you decide which of two risky events you prefer. In a risky event, the outcome is uncertain. The form of the risky events will be described when explaining the stages in-depth.

**Payoff in the experiment**

If this part of the experiment is chosen for payoff, one out of 120 decisions of this part will be randomly selected. The selection will take place at the end of the experiment. For this, a random generator decides which decision will determine your payoff. The draw of each of the 120 decisions occurs with equal probability. Afterwards, the outcome of the chosen risky event will be determined using a random draw which considers the given probabilities. These random draws will be explained in-depth in the descriptions of the particular stages of the experiments.

**Note that only one of the 120 decisions determines your payoff in the experiment and that each of the 120 decisions can determine your entire payoff in the experiment.**

Also note that the risky events can comprise negative outcomes. However, you receive an endowment which can differ between stages. Hence, your payoff is made up of the two components

| **Endowment** and **Outcome of the risky event** |
| --- |

At the end of the experiment each participant will determine his or her relevant decision as well as the outcome of the risky event of this decision by using a random generator on his computer.

**Decision situation**

The risky events displayed in the following figure describe the decision situation you face

in the six stages of the experiment in an abstract way. In each decision situation, you

decide which of the two risky events (here: “Option left” and “Option right”) you prefer.

Up

“Option left“

“Option right“

Down

Up

Down

X

Y

X

Y

and

and

Amount

Amount

Both risky events, “Option left” and “Option right”, comprise a random draw (here Random draw 1) that is depicted as an urn with balls marked “Up” and “Down”. Both Balls in Random draw 1 can be drawn with equal probability. That means, with 50% probability you are in situation “Up” or with 50% probability in situation “Down.”

We now look at the risky event “**Option left**”: If the ball “Up” is drawn, the outcome is X. X can either be a fixed amount or another random draw (Random draw X). If ball “Down” is drawn, the outcome is Y. Likewise, Y can either be a fixed amount or another random draw (Random draw Y).

In risky event “**Option right**”, X and Y follow if the ball “Up” is drawn. In addition, an Amount (blue bank note) is added to both situations “Up” and “Down.” If the ball “Down” is drawn, you receive the Amount indicated on the bank note. If the ball “Up” is drawn, X and Y follow and the Amount (blue bank note) is added. The Amount on the blue bank note can take the following values:

| **-0.50; -0.25; 0.00; ... ; 3.75; 4.00; 4.25** |
| --- |

Hence, for each of these 20 Amounts, one decision situation with two risky events follows. The Amount on the blue bank note is always added to both situations “Up” and “Down” of the risky event where both X and Y occur in situation “Up” (here: “Option right”). Note that on your decision screens, the risky event where the Amount (blue bank note) is added can either be the right or the left option.

**First Stage**

In the first stage of the experiment, you make 20 decisions. You choose on one decision screen at a time which of the two different risky events—Option A or Option B—you prefer.

The risky events can comprise negative outcomes. For each decision in the first stage, you receive an endowment of 25.00. An example of a decision situation in the first stage is provided in the following figure. In this example, the Amount (blue bank note) is added to Option A. The size of the added Amount can be found in the column “**Amount**” on the right-hand side of the screen. For each Amount you decide whether you prefer Option A or Option B.

After activating an Amount in the column “**Amount**”, you decide by clicking on “**A**” or “**B**” whether you prefer Option A or Option B. A grey frame marks the current decision situation. You do not need to stick to a certain order of your decisions. Before you leave the stage, you will have the opportunity to change your decisions.


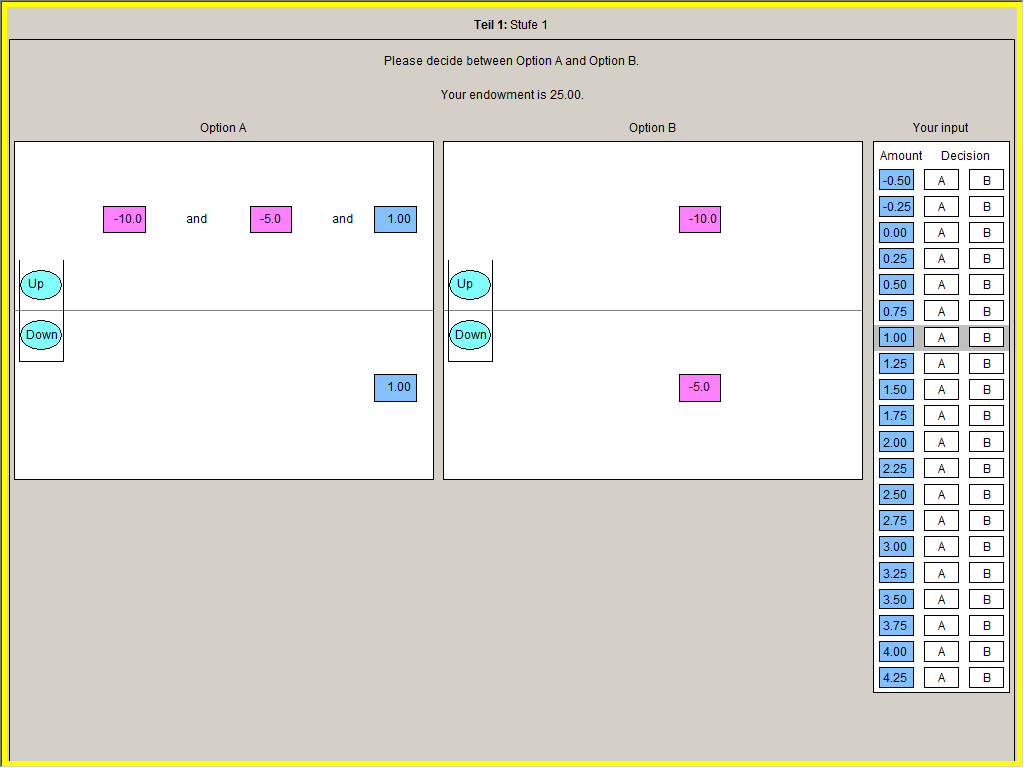


**How is the outcome of the chosen risky event determined in the first stage?** For Random draw 1, there are two balls in an urn – one with the label “Up” and one with the label “Down”. Both balls can be drawn with equal probability.

*Please look again at the example in the previous figure! Suppose this decision has been randomly chosen to determine your payoff.*

*In* **Option A***, the outcome is* -10.00 *plus* -5.00 *plus* 1.00 (Amount on blue bank note), *i.e. in total* -14.00, *if in* Random draw 1 *the ball* “Up” *is drawn. If ball* “Down” *is drawn, the outcome is* 1.00 (Amount on blue bank note). *Under consideration of your* Endowment *of 25.00, your payoff in Option A is* 11.00 *in situation* “Up” *and* 26.00 *in the situation* “Down”.

*In* **Option B***, the outcome is* -10.00 *if in* Random draw 1 *the ball “Up” is drawn. If the ball “Down” is drawn, the outcome is* -5.00*. Under consideration of your* Endowment *of* 25.00*, your payoff in Option B is* 15.00 *in situation* “Up” *and* 20.00 *in situation* “Down”*.*

**Second to Fourth Stage**

In the second to fourth stage you make 60 decisions in total. You choose in three stages, each with 20 decision situations, which of the two different risky events – Option A or Option B – you prefer.

The outcomes of the risky events can be negative. You receive an Endowment of 20.00. An example of a decision situation in the second to fourth stage is provided in the following figure. In this example, the Amount (blue bank note) is added to Option A. The size of the added Amount can be found in the column “**Amount**” on the right-hand side of the screen. For each Amount, you decide whether you prefer Option A or Option B.

After activating an Amount in the column “**Amount**”, you decide whether you prefer Option A or Option B by clicking on “**A**” or “**B**.” A grey frame marks the current decision situation. You do not need to stick to a certain order of your decisions.


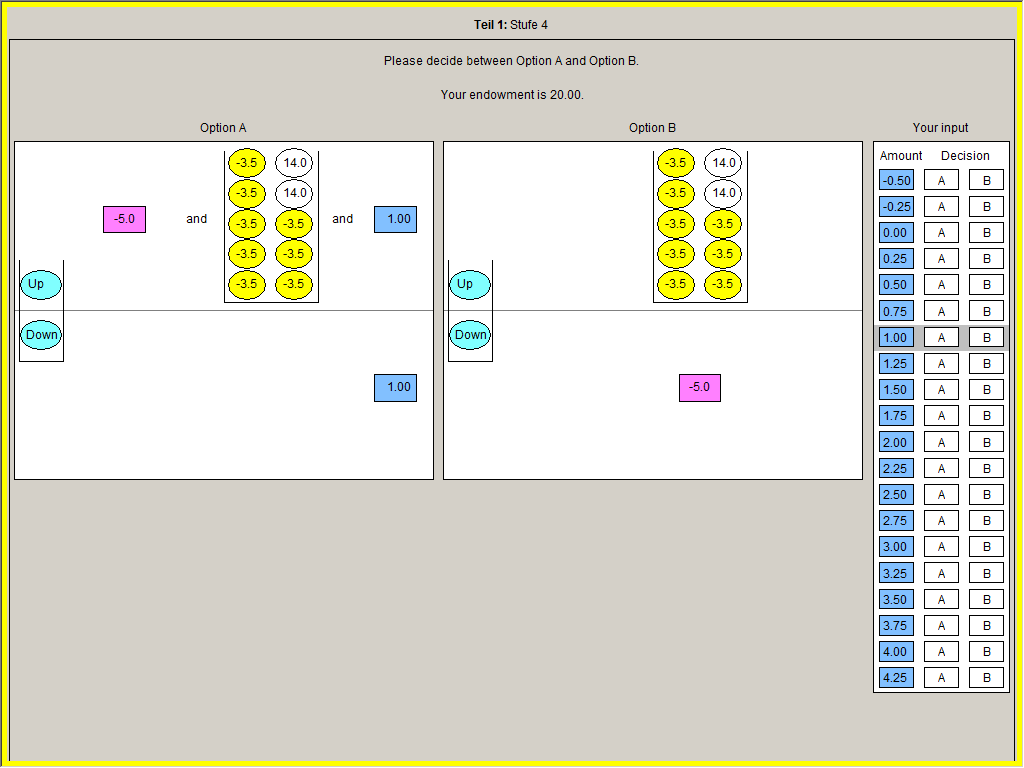


**How is the outcome of the chosen risky event determined in the second to fourth stage?** For Random draw 1, there are two balls in an urn – one with label “Up” an one with label “Down.” Both balls can be drawn with the same probability (analogous to the first stage). As shown in the example in the previous figure, in the second to fourth stage a second random draw (Random draw X) can be necessary to determine your payoff.

In Random draw X, a ball is drawn from an urn containing 10 balls. This ball can either be white or yellow. Note that the composition of white and yellow balls can change between stages two to fourth. This urn always contains 10 balls, and within a stage (with 20 decisions) the composition of white and yellow balls is identical.

*Please look again at the example in the previous figure! Suppose this decision has been randomly chosen to determine your payoff.*

*If in* **Option A** *in* Random draw 1 *ball* “Up” *is drawn, the outcome is* -5.00*, followed by* Random draw X *and an* Amount *of* 1.00 *(blue bank note).*

- *If in* Random draw X *a yellow ball is drawn, you lose* 3.50*. Under consideration of your* Endowment *of* 20.00*, you receive* 12.50 (= 20.00 − 5.00 − 3.50 + 1.00).
- *If in* Random draw X *a white ball is drawn, you receive* 14.00*. Under consideration of your* Endowment*, you receive* 30.00 (= 20.00 − 5.00 + 14.00 + 1.00).

*If in* **Option A** *in* Random Draw 1 *the ball* “Down” *is drawn, the outcome is* 1.00 *(*Amount *on the blue bank note). Under consideration of your* Endowment*,* 21.00 *result.*

*If in* **Option B** *in* Random draw 1 *the ball* “Up” *is drawn,* Random draw X *follows.*

- *If in* Random draw X *a yellow ball is drawn, you lose* 3.50*. Under consideration of your* Endowment *of* 20.00*, you receive* 16.50*.*
- *If in* Random draw X *a white ball is drawn, you receive* 14.00*. Under consideration of your* Endowment*, you receive* 34.00*.*

*If in* **Option B** *in* Random draw 1 *the ball* “Down” *is drawn, the outcome is* -5.00*. Under consideration of your* Endowment*,* 15.00 *result.*

**Fifth and Sixth Stage**

In the fifth and sixth stage you make 40 decisions altogether. You choose in both stages, each with 20 decision situations, which of the two different risky events – Option A or Option B – you prefer.

The outcomes of the risky events can be negative. You receive an Endowment of 17.50. An example of a decision situation in the fifth and sixth stage is provided in the following figure. In this example, the Amount (blue bank note) is added to Option A. The size of the added Amount can be found in the column “**Amount**” on the right-hand side of the screen. For each Amount you decide whether you prefer Option A or Option B.

After activating an Amount in the column “**Amount**” you decide by clicking on “**A**” or “**B**” whether you prefer Option A or Option B. A grey frame marks the current decision situation. You do not need to stick to a certain order of your decisions.


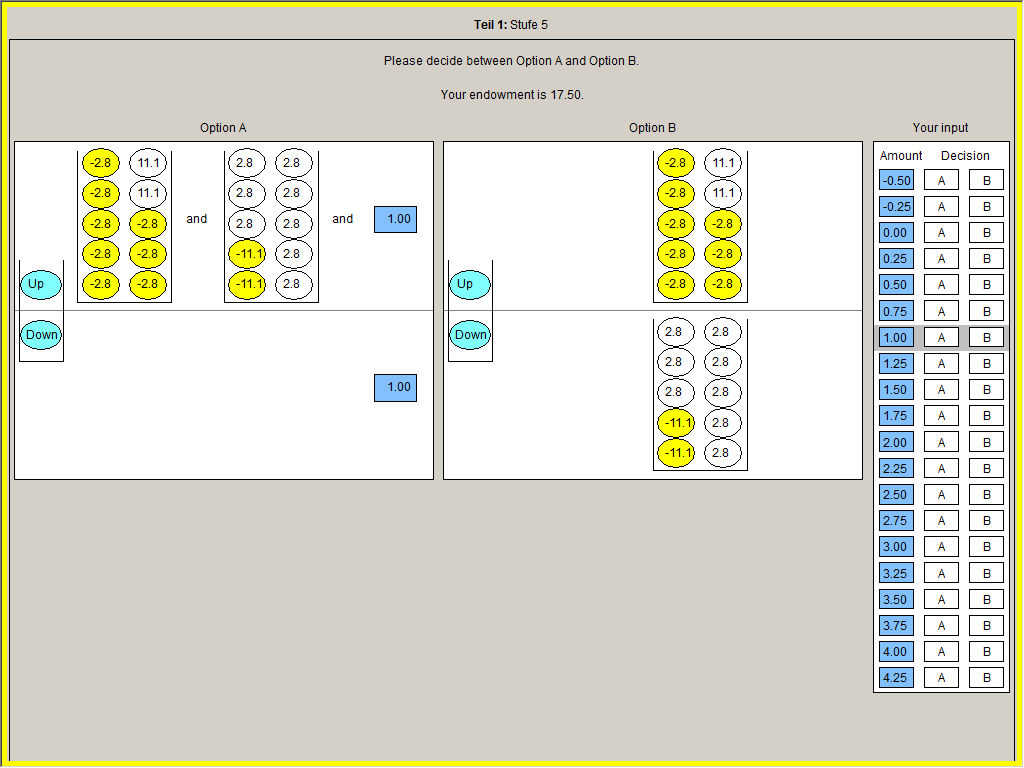


**How is the outcome of the chosen risky event determined in the fifth and sixth stage?** For Random draw 1, there are two balls in an urn – one with label “Up” and one with label “Down.” Both balls can be drawn with equal probability (analogous to the first four stages). As shown in the example in the following figure, in the fifth and sixth stage a second random draw (Random draw X) and / or a third random draw (Random draw Y) can be necessary to determine your payoff.

In Random draw X, a ball is drawn from an urn containing 10 balls. This ball can be either white or yellow. Note that the composition of white and yellow balls can change between the two stages. The urn always contains 10 balls, and within a stage (with 20 decisions) the composition of white and yellow balls is identical. This holds analogously for Random draw Y. Notice, however, that the composition of yellow and white balls across Random draw X and Random draw Y can differ (see figure).

*Please look again at the example in the previous figure! Suppose this decision has been randomly chosen to determine your payoff.*

*If in* **Option A** *in* Random draw 1 *the ball* “Up” *is drawn,* Random draw Y *and* Random draw X *follow. Moreover, the* Amount *of* 1.00 *(blue bank note) is added.*

- *If in* Random draw X *and in* Random draw Y *a yellow ball is drawn, you lose* 2.80 *(from* Random draw X*) and* 11.10 *(from* Random draw Y*). Under consideration of your* Endowment *of* 17.50*, you receive* 4.60 (= 17.50 − 11.10 −2.80 + 1.00).
- *If in* Random draw X *and in* Random draw Y *a white ball is drawn, you receive* 11.10 *(from* Random draw X*) and* 2.80 *(from* Random draw Y*). Under consideration of your* Endowment *of* 17.50*, you receive* 32.40 (= 17.50 + 11.10 +2.80 + 1.00).
- *If in* Random draw X *a white ball and in* Random draw Y *a yellow ball is drawn, you receive* 11.10 *(from* Random draw X*), and you lose* 11.10 *(from* Random draw Y*). Under consideration of your* Endowment*, you receive* 18.50 (= 17.50 + 11.10 − 11.10 + 1.00).
- *If in* Random draw X *a yellow ball and in* Random draw Y *a white ball is drawn, you lose* 2.80 *(from* Random draw X*) and you receive* 2.80 *(from* Random draw Y*). Under consideration of your* Endowment*, you receive* 18.50 (= 17.50 − 2.80 + 2.80 + 1.00).

*If in* **Option A** *in* Random draw 1 *the ball* “Down” *is drawn, the outcome is* 1.00 *(*Amount *on the blue bank note). Under consideration of your* Endowment *you receive* 18.50*.*

*If in* **Option B** *in* Random draw 1 *the ball “*Up*” is drawn,* Random draw X *follows.*

- *If in* Random draw X *a yellow ball is drawn, you lose* 2.80*. Under consideration of your* Endowment *of* 17.50*, you receive* 14.70*.*
- *If in* Random draw X *a white ball is drawn, you receive* 11.10*. Under consideration of your* Endowment*, you receive* 28.60*.*

*If in* **Option B** *in* Random draw 1 *the ball* “Down” *is drawn,* Random draw Y *follows.*

- *If in* Random draw Y *a yellow ball is drawn, you lose* 11.10*. Under consideration of your* Endowment *of* 17.50*, you receive* 6.40*.*
- *If in* Random draw Y *a white ball is drawn, you receive* 2.80*. Under consideration of your* Endowment*, you receive* 20.30*.*

**Before we will start the first part of the experiment, we like to ask you to fill out a comprehension test. For this, please look at the figure on the following page.**

***Figure A***


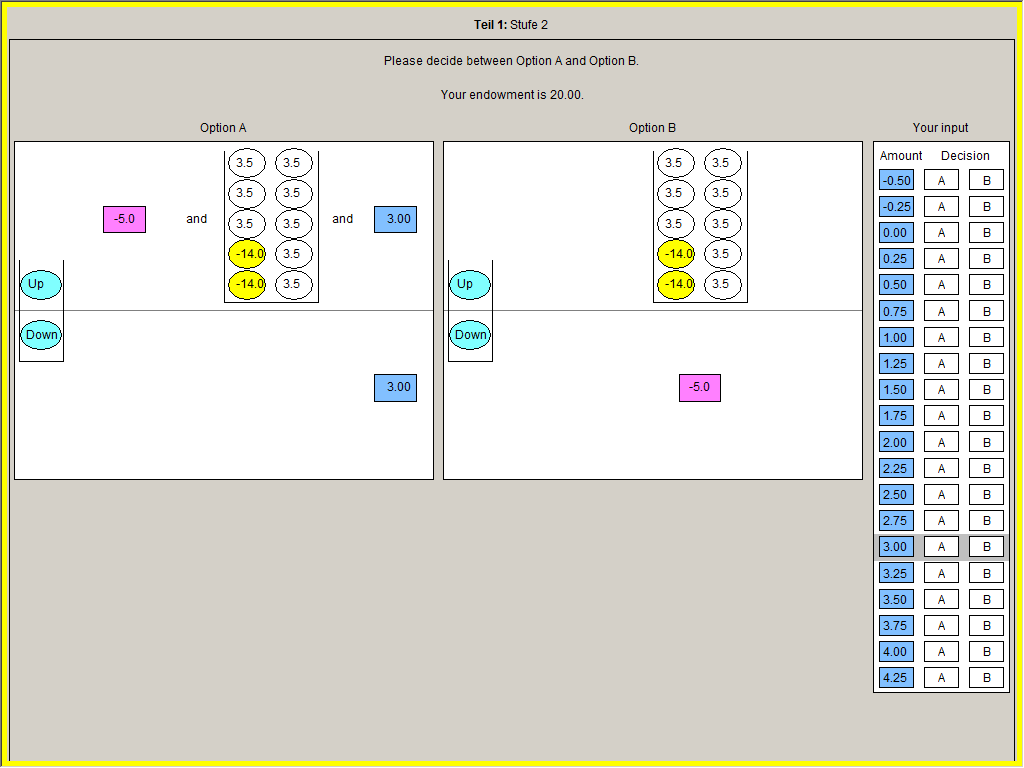


***Figure B***


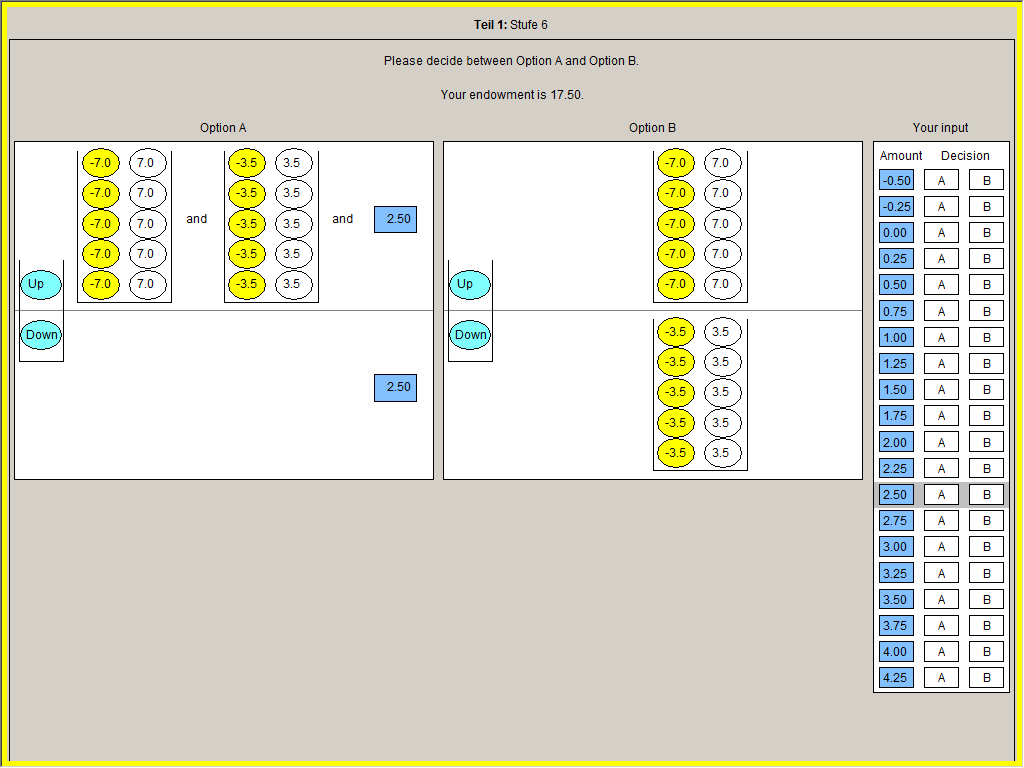


[Comprehension questions were asked on the computer screen and the experiment continued after all questions were successfully answered]

1. **Order of events**

Subjects enter laboratory: Random allocation to workspace

Part I

Risk aversion task

Part II

Randomized Role determination

C

Treatment

O

Treatment

CO

Treatment

Baseline

Treatment

Prudence task

(For each subject randomized order of 3 prudence stages)

Temperance task

(For each subject randomized order of 2 temperance stages)

1 Risk Av. Stage

3 Prud. Stages

(random order)

2 Temp. Stages

(random order)

1 Risk Av. Stage

3 Prud. Stages

(random order)

2 Temp. Stages

(random order)

1 Risk Av. Stage

3 Prud. Stages

(random order)

2 Temp. Stages

(random order)

1 Risk Av. Stage

3 Prud. Stages

(random order)

2 Temp. Stages

(random order)

Questionnaire

Payoff: Task / stage chosen at random, choice executed

Subjects are paid in private and leave laboratory one after another

1. **Instructions Part II**

***Baseline Treatment***

Structure and pay off of the second experimental part are consistent with the first part. The second part is also organized in six stages, where the stages of the second part equal the stages of the first part.

**Note that only one of the 120 decisions determines your payoff in this part and that each of the 120 decisions can determine your entire payoff in the experiment.**

***CO Treatment***

Structure and pay off of the second experimental part are consistent with the first part. The second part is also organized in six stages, where the stages of the second part equal the stages of the first part.

In contrast to part 1, decisions are made for a team of two players in part 2. This means that the choices taken determine the payoff of both players

Decisions will be entered only from one team member (player 1). The other team member (player 2), however, will see the decisions on the computer screen. You will see on the decision screen which player you are.

You will have the opportunity to exchange messages with the other player via chat. The chat will be shown on the computer screen to your right which will be switched on for this purpose. The content of the chat is – in principle – of your choice. It is not permitted, however, to share personal information like name, age, address, subject of study (this includes references to teachers, courses or course contents that enables the identification of the subject of study), or similar. Violations of the communication rules will lead to the exclusion from the experiment and to no payment.

**Note that only one of the 120 decisions determines your payoff in this part and that each of the 120 decisions can determine your entire payoff in the experiment.**

***O Treatment***

Structure and pay off of the second experimental part are consistent with the first part. The second part is also organized in six stages, where the stages of the second part equal the stages of the first part.

In contrast to part 1, decisions are made for a team of two players in part 2. This means that the choices taken determine the payoff of both players

Decisions will be entered only from one team member (player 1). Meanwhile, Player 2 enters the decisions he expects player 1 is making (however, the entered expectations from player 2 will have no impact on the pay offs of both players). You will see on the decision screen which player you are.

**Note that only one of the 120 decisions determines your payoff in this part and that each of the 120 decisions can determine your entire payoff in the experiment.**

***C Treatment***

Structure and payoff of the second experimental part are consistent with the first part. The second part is also organized in six stages, where the stages of the second part equal the stages of the first part.

In contrast to part 1 you will have the opportunity to exchange messages with another player via chat. However, your will still decide for yourself. Choices of your chat partner will not impact your own pay offs.

The chat will be shown on the computer screen to your right which will be switched on for this purpose. The content of the chat is – in principle – of your choice. It is not permitted, however, to share personal information like name, age, address, subject of study (this includes references to teachers, courses or course contents that enables the identification of the subject of study), or similar. Violations of the communication rules will lead to the exclusion from the experiment and to no payment.

Please note that subsequent stages will be activated after your chat partner completed his choices in the current stage. The „Next“-button will appear not till then.

**Note that only one of the 120 decisions determines your payoff in this part and that each of the 120 decisions can determine your entire payoff in the experiment.**

1. **Individual choice patterns**

The following figures displays the choice patterns of decision making individuals *i* in the four treatments for the risk aversion, prudence and temperance tasks. Each point within a figure indicates an individual’s average premium in Part I ($\bar{m}_{i}^{I}$) and his average premium in Part II ($\bar{m}_{i}^{II}$*^I^*). Each combination of premia ($\bar{m}_{i}^{I}$*,* $\bar{m}_{i}^{II}$) is either displayed as triangle or a square. When the partner’s choices in Part I resulted in a larger average premium the triangle is displayed, otherwise the square is shown. Points above the dashed line indicate an increase in risk premia from Part I to II while points below indicate a decrease. If individuals consider the preferences of the partners they are matched with, we expect triangles to appear above the dashed line and squares below. As the regression results would suggest, this pattern is most pronounced for the CO treatment in Figures E1 and E2.

**Figure E1: Influence of partners on individual decisions in Part II (risk aversion)**

**Figure E2: Influence of partners on individual decisions in Part II (prudence)**

**Figure E2: Influence of partners on individual decisions in Part II (temperance)**

1. **Communication between decision-making individuals and partners**

On average, the subjects in the two communication treatments C and CO of the Shift sessions exchange 35 messages with an average of 29 characters in length over the course of the six stages of Part II. A pair of subjects writes around six more messages in the CO treatment than in the C treatment (*p* = 0.029, two-sided Mann-Whitney-*U* test) but the sum of characters written does not differ significantly (*p* = 0.195). In neither treatment does the number of messages nor the number of characters differ between individuals and partners (*p* ≥ 0.217, two-sided Wilcoxon tests).

To gain some first insights on the role communication plays in influencing decisions in social settings, we classify the messages sent by individuals and their partners based on content. Two research assistants independently coded all of the messages according to a simple classification scheme. We present brief results on the two following content categories:

1. Does the subject mention his preferred choice?
2. Does the subject express agreement with the other’s preferred choice?

Both were coded as binary variables (*yes* or *no*) message by message and were classified consistently (Krippendorf’s Alpha ≥ 0.728). ^^[[1]](#footnote-1)^^

First, we compare the message content between the pairs of the two treatments. Even though there are ten messages on average in CO that cover the sender’s preferred choice and only seven in C, the number of messages and the number of characters does not differ significantly between the two treatments (*p* ≥ 0.106, two-sided Mann-Whitney-*U* tests). In C 81% of the pairs mention a preferred choice at least once. In CO this share is 83%. Agreement with the other subject’s preferred choice is significantly more often voiced in the CO treatment than in the C treatment (*p* ≤ 0.002). In CO on average seven messages contain this content; in C three messages mention agreement. Agreement with the other’s choice is voiced at least once in 54% percent of the pairs in C but in all teams of CO.

Second, we compare the message content across roles within the two treatments. As one might expect, there are no differences between roles in the C treatment because both roles are symmetric (*p* ≥ 0.262, two-sided Wilcoxon tests). In the CO treatment, we do not find any differences with respect to the number characters used to cover the two topics (*p* ≥ 0.323). However, the passive partners send on average two more messages mentioning their own preferred choice than do the decision making individuals (*p* = 0.013). They also send two and a half messages more expressing their agreement with the other’s preferred choice (*p* = 0.001).

1. In a first meeting the research assistants met with one of the authors who explained the data set and the classification scheme to them. They then classified a training sample based on the observations excluded from the CO treatment. The classification discrepancies and remaining questions were discussed in a second meeting. After that, the research assistants coded the complete data set independently. Any questions arising during the classification process were answered over a shared mailing list by one of the authors. Note that we coded for additional content but none of the other categories passed the usual consistency threshold of a Krippendorf’s Alpha of 0.7. [↑](#footnote-ref-1)
